# Evaluation of predictive capability of Bayesian spatio-temporal models for Covid-19 spread

**DOI:** 10.1186/s12874-023-01997-3

**Published:** 2023-08-11

**Authors:** Andrew B. Lawson

**Affiliations:** 1 Division of Biostatistics and Bioinformatics, Department of Public Health Sciences, Medical University of South Carolina, 135 Cannon Street, Charleston, 29425 USA; 2 School of Medicine, Usher Institute, University of Edinburgh, Edinburgh, UK

**Keywords:** Covid-19, Pandemic, Spatio-temporal, Bayesian hierarchical modeling (BHM), MASE, MAE, MSE, Retrospective, Prospective

## Abstract

**Background:**

Bayesian models have been applied throughout the Covid-19 pandemic especially to model time series of case counts or deaths. Fewer examples exist of spatio-temporal modeling, even though the spatial spread of disease is a crucial factor in public health monitoring. The predictive capabilities of infectious disease models is also important.

**Methods:**

In this study, the ability of Bayesian hierarchical models to recover different parts of the variation in disease counts is the focus. It is clear that different measures provide different views of behavior when models are fitted prospectively. Over a series of time horizons one step predictions have been generated and compared for different models (for case counts and death counts). These Bayesian SIR models were fitted using MCMC at 28 time horizons to mimic prospective prediction. A range of goodness of prediction measures were analyzed across the different time horizons.

**Results:**

A particularly important result is that the peak intensity of case load is often under-estimated, while random spikes in case load can be mimicked using time dependent random effects. It is also clear that during the early wave of the pandemic simpler model forms are favored, but subsequently lagged spatial dependence models for cases are favored, even if the sophisticated models perform better overall.

**Discussion:**

The models fitted mimic the situation where at a given time the history of the process is known but the future must be predicted based on the current evolution which has been observed. Using an overall ‘best’ model for prediction based on retrospective fitting of the complete pandemic waves is an assumption. However it is also clear that this case count model is well favored over other forms. During the first wave a simpler time series model predicts case counts better for counties than a spatially dependent one. The picture is more varied for morality.

**Conclusions:**

From a predictive point of view it is clear that spatio-temporal models applied to county level Covid-19 data within the US vary in how well they fit over time and also how well they predict future events. At different times, SIR case count models and also mortality models with cumulative counts perform better in terms of prediction. A fundamental result is that predictive capability of models varies over time and using the same model could lead to poor predictive performance.

In addition it is clear that models addressing the spatial context for case counts (i.e. with lagged neighborhood terms) and cumulative case counts for mortality data are clearly better at modeling spatio-temporal data which is commonly available for the Covid-19 pandemic in different areas of the globe.

**Supplementary Information:**

The online version contains supplementary material available at 10.1186/s12874-023-01997-3.

## Background

Bayesian models have been applied throughout the Covid-19 pandemic especially to model time series of case counts or deaths (e.g [1, 2]; MedRxiv repository: https://connect.medrxiv.org/relate/content/181). Fewer examples exist of spatio-temporal modeling, even though the spatial spread of disease is a crucial factor in public Health monitoring [3]. The ability of infectious disease (ID) models to estimate the true disease risk is important in the assessment of the relevance of the modeling approach. Equally, the ability of models to predict the future behavior of the ID is clearly also important for public health (PH) planning. It is also clear that models that provide good goodness of fit (GOF) do not always provide good predictive ability. Some evaluation of predictive capability of Covid-19 models has been attempted previously. Ensemble models (see e.g. https://covid19forecasthub.org/doc/ensemble/) which accumulate results from a range of models were found to perform reasonably well compared to individual models for case [4], mortality [5], and hospitalisation [6]. However there has also been criticism of previous model prediction attempts (see eg [7]). Often the prediction is for marginal time series rather than the spatio-temporal joint distribution. The importance of examining the complete spatio-temporal dynamic of an epidemic has been stressed previously [7]. Some recent approaches have also addressed space-time in ID modeling [8, 9]. While various time horizons are often evaluated for prediction accuracy, it is often clear that accuracy degrades quickly with extensive horizons [4]. The greatest accuracy is confined to single steps. In this paper an evaluation of the behavior of one step predictions from different spatio-temporal models are assessed and reported.

## Methods

### Data

**Fig. 1:**
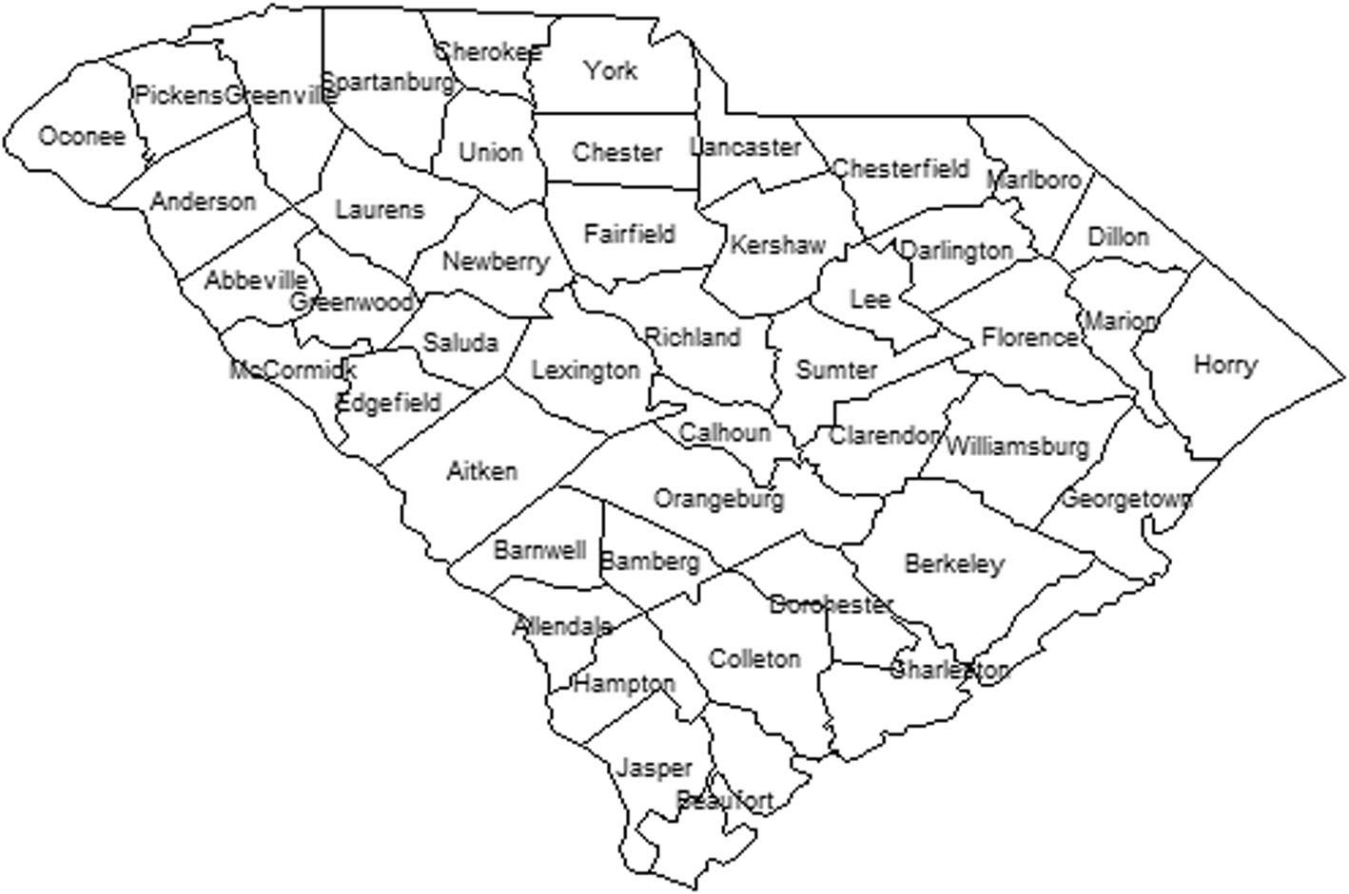
County level map of South Carolina

Here data from the US state of South Carolina is examined at the county level. There are 46 counties in this state (see Fig. 1). Health policy is managed statewide and so Covid-19 response is at the state level. Whilst some cross border movement between states is possible the statewide administration of health policy limits cross boundary effects. Here we examined case counts and deaths at the county level.

Our case count data (number of positive tests) at the county level is obtained from Department of Health and Environmental Control (DHEC) in South Carolina, while the mortality count data is obtained from the National Center for health Statistics (NCHS) as reported in https://github.com/nytimes/covid-19-data. The data is publicly available at this site and also available at JHU Hub site (https://github.com/CSSEGISandData/COVID-19). The reason for using the NYT site is that the derivation of the data are described in greater detail.

**Fig. 2:**
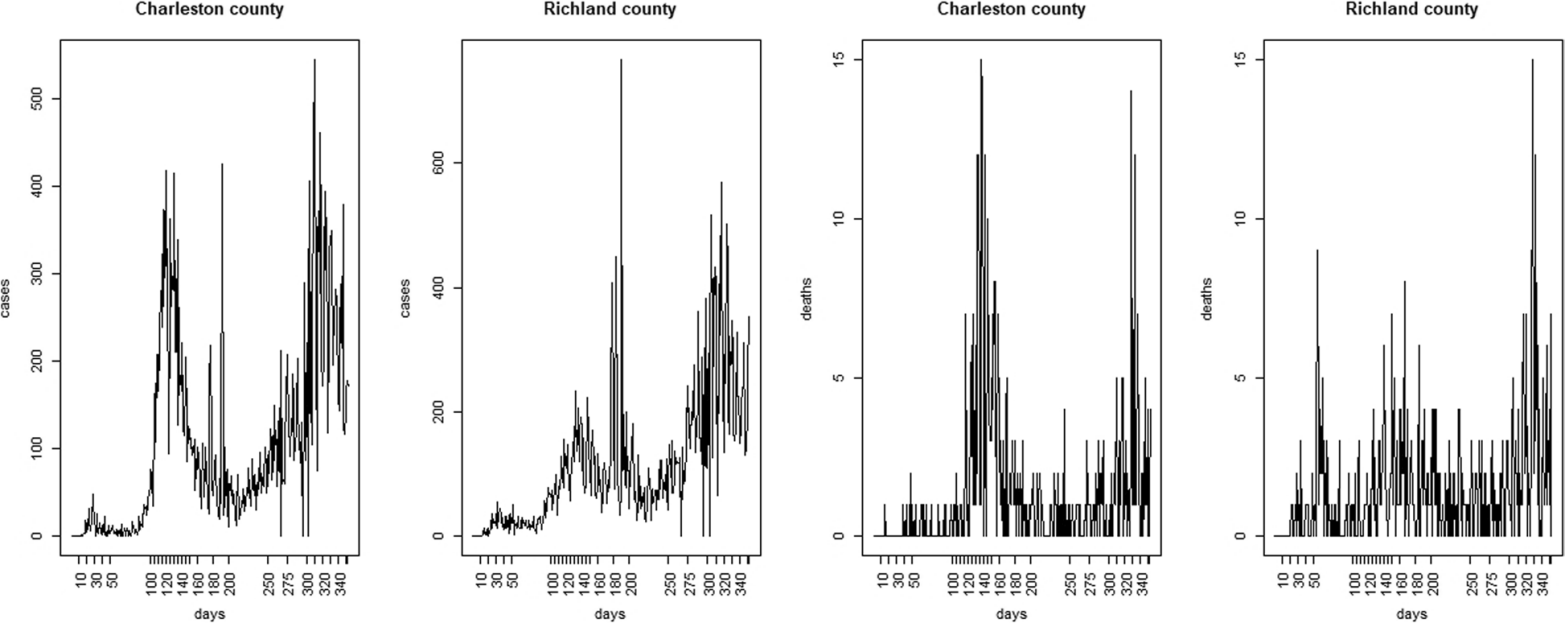
Daily case count of Covid-19 positive tests (left) and deaths (right) for Charleston and Richland counties SC, during the three waves in 2020–2021

Figure 2 displays the distribution of case counts and deaths for Charleston and Richland Counties and is typical of 46 counties within the state of South Carolina. The timing of different waves and their characteristics vary across counties of course. The time range is March 6^th^ 2020 to February 21^st^ 2021, which encompasses the main three waves of infection but excludes the main effect of vaccination, from March 2021 onwards.

### Model development and fitting

In previous studies, a range of models have been fitted to these data. First an initial period in 2020 was examined [10] and later the full 353 day period [11]. Models were fitted retrospectively over the complete time period. The Bayesian models were fitted using Markov chain Monte Carlo (McMC) using the R package Nimble. Convergence was checked using the R package CODA. Time to convergence, which varied between models, was always after 20,000 iteration burn-in, with sample size of 10,000. A model comparison was made based on goodness of fit using the Watanabe Akaike Information Criterion (WAIC). The WAIC is a standard relative goodness-of-fit criterion and is now replacing the commonly used deviance Information criterion (DIC) as it can be stably estimated within McMC applications [12]. It is also closely related to leave-one-out cross-validation. In the later study [11], it was found that a Susceptible-Infected-Removed (SIR) model in discrete time with dependence on lagged neighborhood counts and a set of deprivation predictors was the best fit for the case count data (model 5A). For the mortality data dependence on case count and cumulative case counts was also the best model.

Define case count $y_{ij}$ in the *i* th area and *j* th time period, for *m* areas and *J* time periods, with susceptible population $S_{ij}$: and $y_{ij} \sim Pois(\mu_{ij} ){\text{ with }}\mu_{ij} = S_{ij} .f(y_{i,j - 1} ,........)$

where $p_{i,j - 1}^{{}} = \log f(y_{i,j - 1} ,........)$ is a propagator which can be flexibly modelled to allow for variation in transmission in time and space. In the models developed in [11] the lowest WAIC model was found to have a propagator of the form:

$$p_{i,j - 1}^{{}} = \alpha_{0} + \alpha_{1} log(y_{i,j - 1} ) + \alpha_{2} \log (\sum\limits_{{k \in \delta_{i} }} {y_{k,j - 1} )} + v_{i} { + }x_{i}^{t} \beta \,$$

In this model, there is dependence on previous count in the same region ($y_{i,j - 1}$) and also the sum of previous counts in neighboring regions ( $\sum\limits_{{k \in \delta_{i} }} {y_{k,j - 1} }$). In addition, a set of three deprivation predictors (% black population, % under the poverty line, multi-dimensional deprivation index 2017 based on the American Community Survey) were included in a linear predictor ($x_{i}^{t} \beta$) and were found to significantly increase explanation. Finally, an uncorrelated random effect ($v_{i}$) was also found to be important. The effect of asymptomatic transmission is included via a scaling effect on the previous count. While estimates of the rate varied during the pandemic, from around 50% to 17%, the most recent estimates suggest a low percentage [13]. A rate of 20% has been used in this work, and this appeared to provide the best overall fit. This model is denoted as the Full model in later discussion.

The case count model is completed by an accounting equation which updates the susceptible population: $S_{ij} = S_{i,j - 1} - y_{i,j - 1} - R_{i,j - 1}$, with $y_{i,j - 1}$ replacing the true infective count, which is unobserved. Removal is assumed to occur at a given rate based on infectives and deaths i.e. $R_{i,j} = \rho y_{i,j - 1} + d_{i,j - 1}$, where $d_{i,j - 1}$ is the observed death count in the *i* th region and previous time period.

In the previous analysis, deaths are assumed to be given and have not been modelled. However to make any predictions from these models, mortality must be modelled. The best fitting mortality model for the SC county level deaths, as found in many other studies, was based on both current case load and cumulative case counts:

$$\begin{gathered} d_{i,j} \sim Pois(\mu_{ij}^{d} ) \hfill \\ \log (\mu_{ij}^{d} ) = \alpha_{0}^{d} + \alpha_{1j}^{d} \log (y_{i,j} ) + \alpha_{2j}^{d} \log (T_{i,j - 1} ) + v_{i}^{d} \hfill \\ where \, T_{ij} = \sum\limits_{k = 1:j} {y_{i,k} } \hfill \\ \end{gathered}$$

and $v_{i}^{d}$ is an uncorrelated county level random effect. The superscript *d* denotes death parameter. This model is denoted as Full model. Whereas a death count model with the cumulative count removed is denoted aa the Base model in later comparisons.

In the above models all the regression parameters and random effects have zero mean Gaussian prior distributions with precisions assumed to have weakly informative gamma prior distributions: Ga(2,0.5). In what follows, one step ahead predictions have been made for case counts and deaths with modelled mortality.

### Predictive measures

The assessment of predictive capability of models is an important focus. Here the focus is on the temporal prediction beyond the current available data. Note that one-step predictions are assessed only as longer time horizons can lead to considerable degeneration in prediction due to the stochastic nature of the SIR formulations, see eg [14].

To assess the predictive capability of the model-based analysis, it is possible to use different rules for scoring loss [15, 16]. Of these, proper scoring rules are often favored. In the following we use a range of proper scoring rules based on squared error and absolute error loss. While these loss functions are optimal for Gaussian-type observations, they are generally robust to departure from the Gaussian assumptions [15]. Our strategy was to make one step ahead predictions at various time points during the 3 epidemic waves. Models were fitted up to the time point and then a one-step prediction made. This was done to mimic the way that prospective analysis of an infectious disease would be made at different times during the pandemic. It is likely that different epidemic periods could lead to divergence in the accuracy of predictions. The time points chosen for the evaluation were, in days:

10,20,30,40,50,100,105,110,115,120,125,130,135,140,145,150,160,180,200,250,275,300,310,320,330,340,350,352. These were chosen to represent different aspects of the time variation. In the analysis reported here a model is fitted retrospectively at a given time point and then a one step prediction is made and compared to the observed case count or death count at the next time. At each of the 28 time points a full McMC model fit was performed. The difference between the prediction and observed is the focus of our prediction metrics.

Define the following at time T:

$y_{i}^{P} , \, d_{i}^{p}$ one step prediction of case and death count at T + 1, and the case loss is.

$e_{i} = y_{i}^{p} - y_{i,T + 1}$ and the death loss as $e_{i}^{d} = d_{i}^{p} - d_{i,T + 1}$. Then the following summary metrics were computed for case counts:

$$c_{1i} = \sum\limits_{k = 2:T} {abs[y_{i,k} } - y_{i,k - 1} ]/(T - 1)$$

and $ASE_{i} = abs(e_{i} /c_{1i} )$ which is the absolute scaled error [17],

$APE_{i} = abs(e_{i} )$ which is the absolute error of the prediction. Taking means over the areas this leads to MASE, MAPE. The mean squared error (MSE) was also considered but yielded results similar to the MAPE in overall form and is not discussed in detail for brevity. Note that these are all predictive loss measures. Once the metrics are computed for a particular model then model differences can be assessed. For death counts we computed the MSE and MAPE only.

## Results

A total of 28 McMC runs were made to produce the one step predictions and loss measures for any given model. Here we compare the difference in predictive capability of a simple SIR model to the optimal retrospective model which was previously reported (Full model). The base SIR model (denoted ‘Base model’) had a propagator of the form:

$p_{i,j - 1} = \alpha_{0} + \alpha_{1} log(y_{i,j - 1} ) + v_{i} \,$ with only a temporal dependence on previous cases in the same area, no dependence on previous neighbors and only a uncorrelated regional effect.

**Fig. 3:**
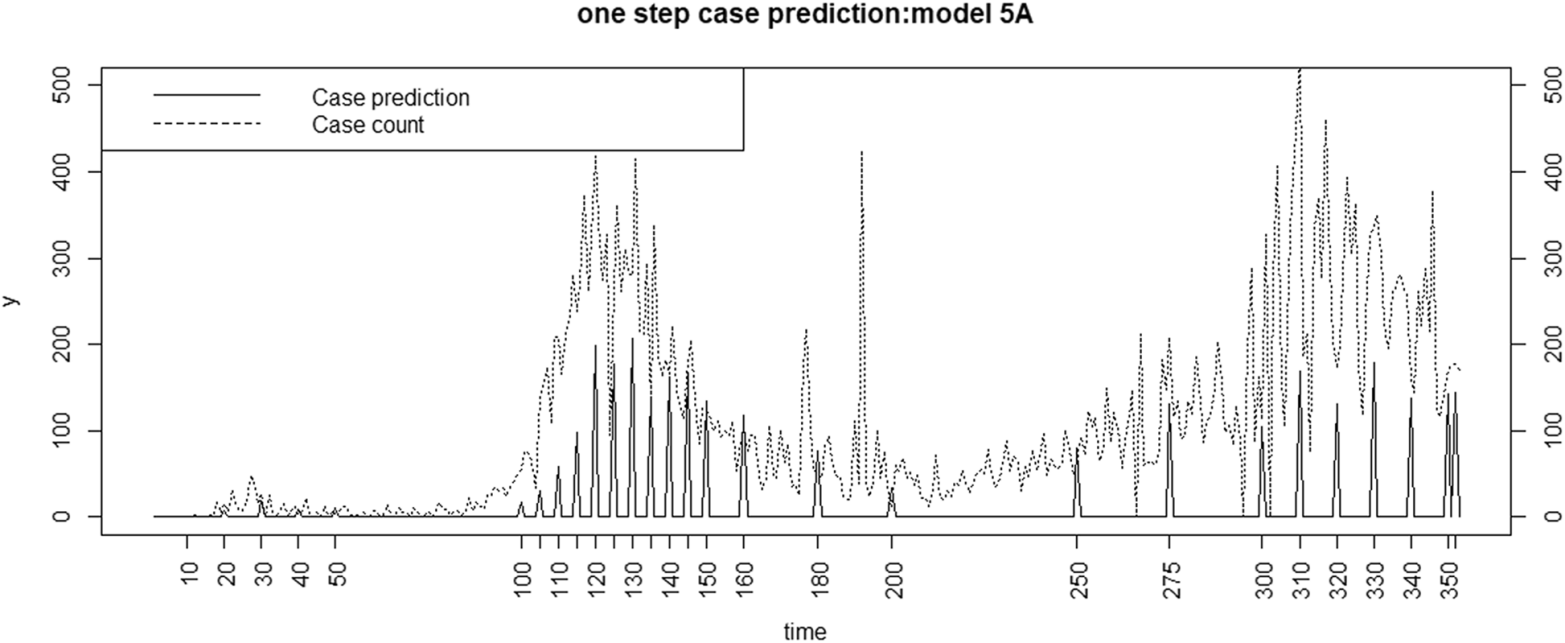
Time series plot of case count against one step prediction at 28 time points for Full model for Charleston county

**Fig. 4:**
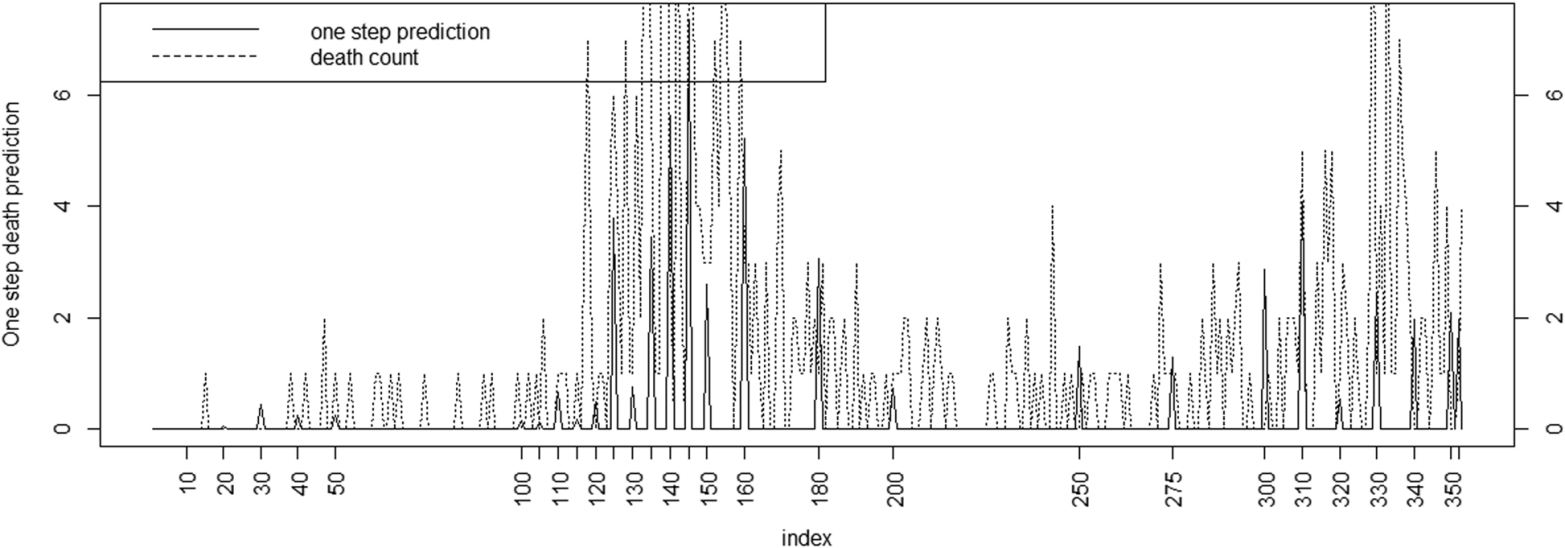
Time series plot of death counts against one step prediction at 28 time points for Full model for Charleston county

Figure 3 displays the one step case count prediction, compared to the observed count for Charleston county. Figure 4 displays the death count prediction for the same region.

**Fig. 5:**
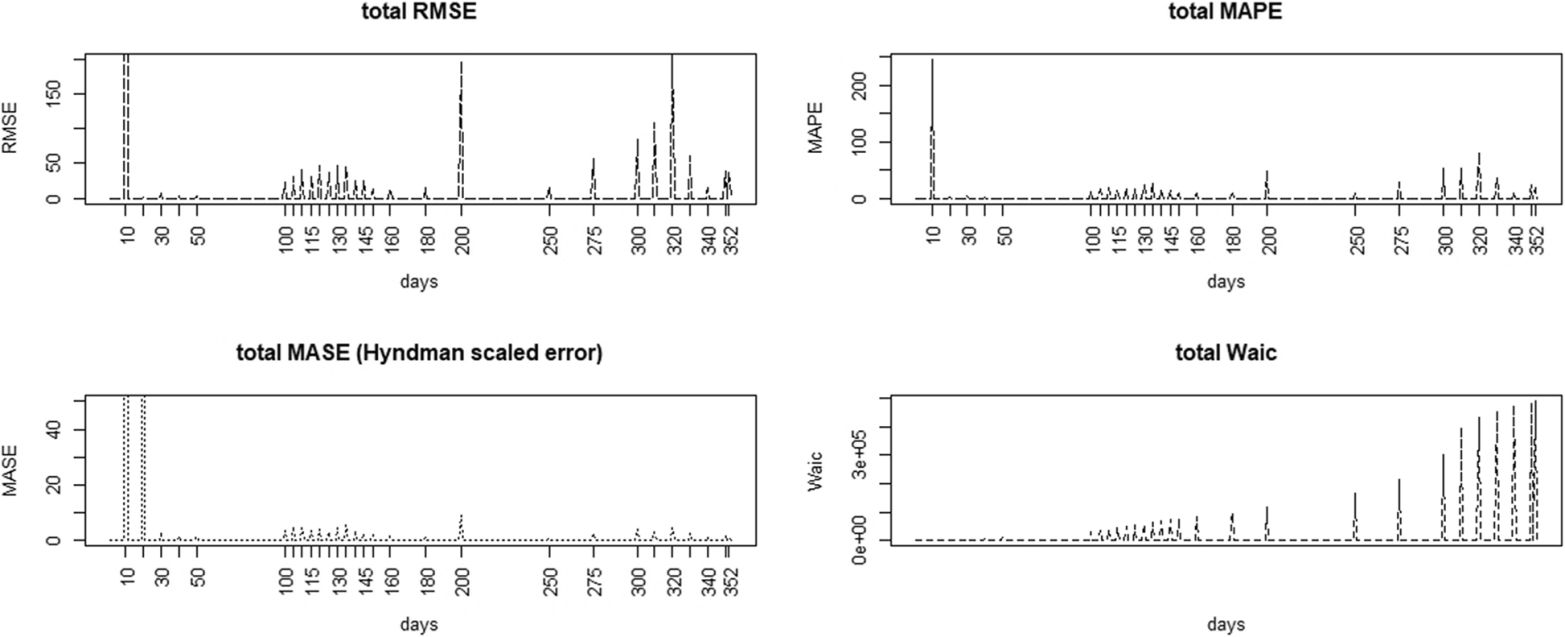
Total RMSE, MAPE, MASE, and WAIC for case count Full model (row-wise from top left)

Figure 5 Displays the a multi panel plot of the case count three predictive mesures and WAIC for Full model averaged over the spatial regions. Note that the WAIC increases over time due to the change in the size of observation set. It is clear that the case count MSE and MAPE tend to follow similar patterns with the root MSE (RMSE) displaying larger spikes of loss at certain points. The MAPE and MASE display somewhat similar behavior overall. Its also notable that the main divergences of prediction are at peak times during the outbreak. This suggests that predictions during waves will be less reliable

**Fig. 6:**
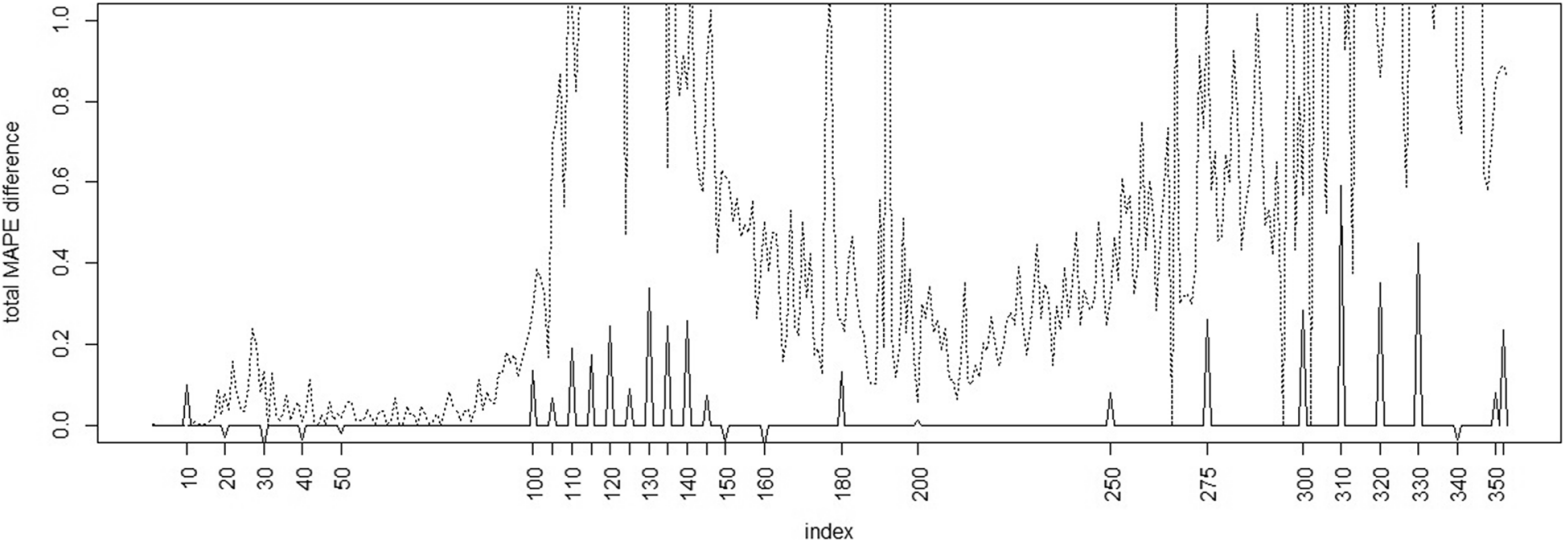
Time series profile of total difference in MAPE between Full model and Base model (with Charleston county case count superimposed)

Figure 6 displays the total MAPE difference between case count Full model and Base model.

**Fig. 7:**
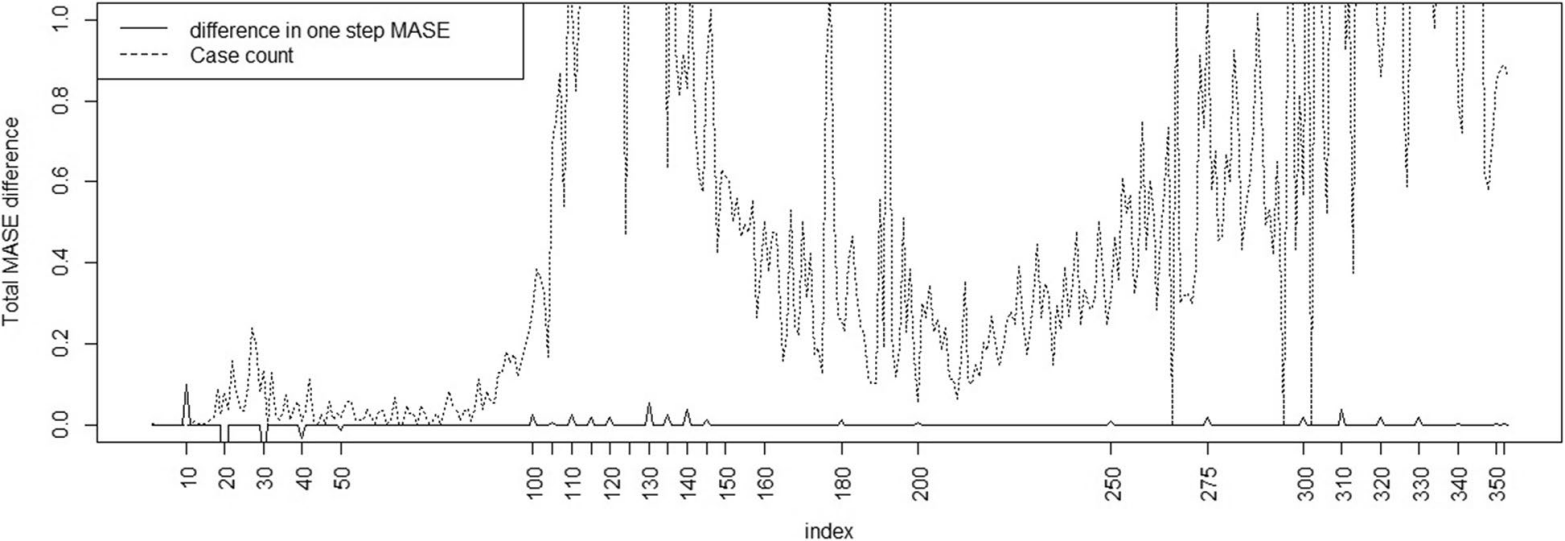
Total MASE difference between Full model and Base model (with Charleston county case count superimposed)

In terms of MAPE it is noticeable that during the first wave the simple model appears to have better prediction accuracy (i. e. lower loss) than Full model. However this is not the case for later waves and after time 100 there is mostly lower loss (and better prediction) from the Full model. This is also particularly true during the main wave peaks. In addition, the MASE difference mirrors this behavior and only shows better prediction for the Base model in the first wave only. Figure 7 displays the total difference for the MASE between the Base and Full model.

**Fig. 8:**
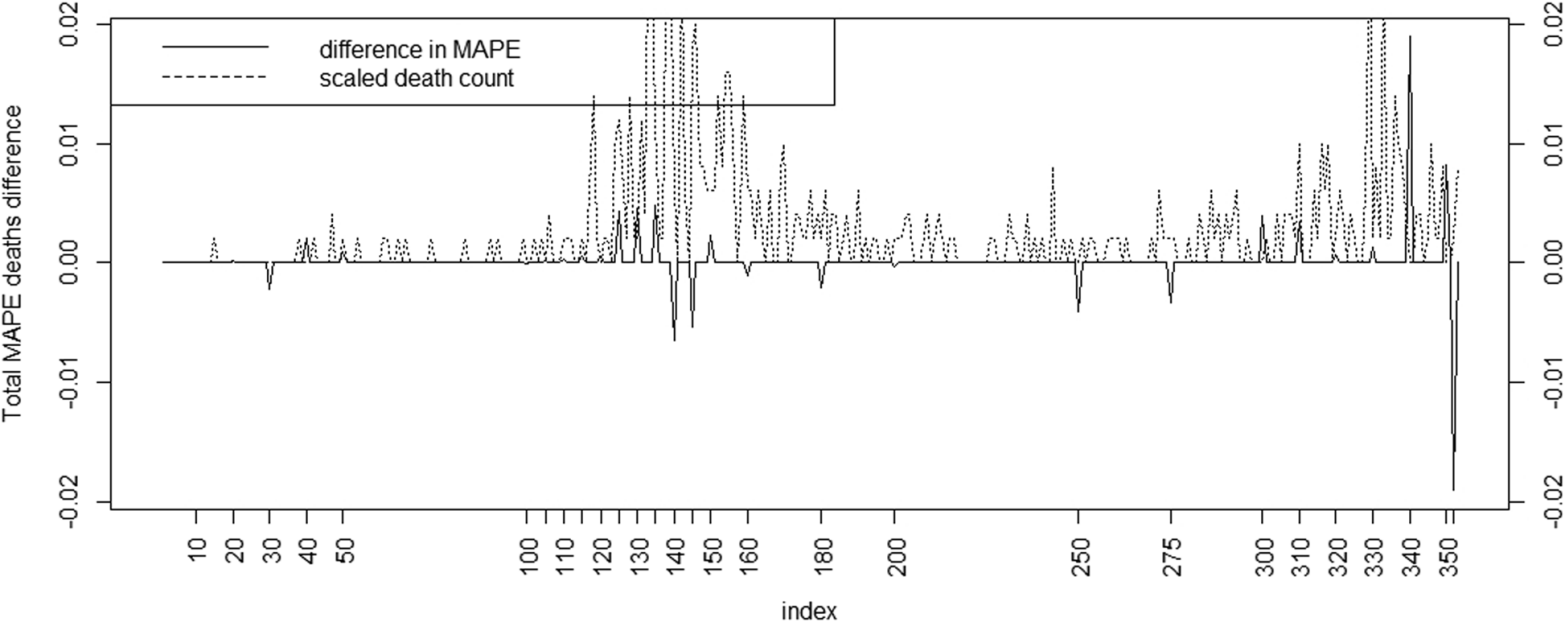
Total MAPE difference for death counts for Base and Full model (with Charleston county death count superimposed)

Figure 8 displays the death count prediction difference for the MAPE, where the full death model dependent on cumulative cases is compared to a Base model with only current case count dependency. Similar performance is found for the MSE for death counts (see Additional file 1: Appendix Table 1) and is not included here for brevity.

Inspection of Fig. 8 demonstrates that the prediction quality is not maintained during the second wave and this is borne out in the MAPE difference which displays variable performance between the two models during that wave. The best death model with cumulative case count dependence seems to perform better at the beginning of waves, but due to the highly variable death counts this is not maintained.

Additional file 1: Appendix Table 1 displays a selection of 14 days and the average results over the state for the metric differences for the chosen models. The days chosen are meant to represent the different periods where there are particular changes in pandemic activity.

It is clear that in the early first wave (as represented by days 20—50) the count MAPE and MASE are lower for the simpler models. This is also true for the death MAPE but the difference is less marked. By day 100 the overall MAPE, MASE and death MAPE are lower for the best model (case Full model and death model with cumulative count dependence). By day 310 this is also the case but by day 350 shows a lower difference. Overall the case count measures seem to follow similar trajectories. The first day times are similar. For the death count MAPE and MSE the picture is more variable. The Full model appears favored at the beginning of the first wave and at around day 300 but alternates at other times. While displayed results for a single county within a single US state are superimposed on the statewide average here (Figs. 6, 7 and 8), the results are comparable across many counties.

## Discussion

This paper is an attempt to evaluate the predictive ability of models that can be used for Covid-19 case counts and mortality. There are a number of important results which have arisen in this work. First, it is clear that during the early stages of the pandemic, simple case time series models (without neighborhood lagged effects) and simple death count models do better in one-step prediction.

It is to be expected that in the early stages of an outbreak, it is likely to be confined in spatial extent and limited in its transmission. The effects of deprivation in addition may not be expressed clearly in the initial stages of an outbreak. In addition, a data quality issue may clearly confound the early recording of case and death counts, and this under-ascertainment could vary over time.

Without, the benefit of having ‘test positive’ and ‘test negative’ data it is not possible to clearly assess the degree to which the population spread of the virus is occurring. In addition without serological surveys it is not possible to clearly assess the asymptomatic transmission, at any given time. In fact repeated surveys would have to be carried out to do so. The published literature during the pandemic has different estimates for asymptomatic transmission and in [11] it was decide that a 20% rate would be a suitable balance estimate.

During the main wave peaks of the pandemic the lagged effect models with neighborhood effects for case counts and the cumulative count models for mortality perform better than the simple alternatives examined here. However it is also true that during the peak waves of the pandemic the ‘best’ models do not provide such good predictions compared to non-peak periods. Figs 3 and 4 demonstrate this effect. In fact this appears often to be the case with a range of epidemic models in the literature (see e.g. [18] suppl Fig. 2, or [19] for an examples). The result is that while it is possible to model peak behavior very closely, it usually requires the use of time dependent random effects ([11, 20] Fig. 4), and so the predictive capabilities of such models are limited due to the overfitting.

Some short comings of the work are apparent. First, although the models reported here have been validated in at least two US states at county level and in the UK, alternate models could be found to be evaluated and so this evaluation is only limited to those chosen. For example, models using reproduction numbers and unobserved (latent) exposed groups are sometimes suggested. These models have identification issues and so have been avoided here. Second, I have not made any probabilistic assessment of the major differences between the derived prediction metrics and this could be an important future direction. It has also not been a focus to look at longer predictions beyond one-step. This can be a difficult task with SIR models as they become degenerate without added injections of risk (such as new variants or jump diffusions). As jump diffusions are not simple to predict, then it is difficult to make good predictions of where and when any future wave may occur. On the other hand we have examined the effect of change to prior distributions for parameters and their effect on estimation. In particular we varied the precision prior distributions for regression parameters and random effects. This did not affect the estimation of the models to any significant degree and the predictions were robust to these sensitivity changes.

## Conclusions

Finally it is important that the context of the model fitting and prediction is stressed. In this report, models which were compared for their retrospective goodness of fit to three waves of pandemic data were assumed in the predictive comparisons. During a pandemic the best model would not be known in advance and so judgements may be made based on ‘best available‘ modeling. Initially simple models are likely to be considered, given available data. Later more sophisticated models, possibly within ensembles, are likely to useful. In our analysis we have found that this is supported by the preference for simple predictive models initially and lagged neighborhood models later. The basic fact that model performance varies during the pandemic is an important fundamental takeaway from these performance assessments. The fact that the best models for later stages of the pandemic include neighborhood effects is also an important aspect of the predictive modeling that should be taken on board: Covid-19 spread is spatial as well as temporal in nature.

### Supplementary Information

**Additional file 1. **

## Data Availability

The data is publically available from the NYT GitHub site at https://github.com/nytimes/covid-19-data
