# Supplementary material for: Evaluation of predictive capability of Bayesian spatio-temporal models for Covid-19 spread
Source: BMC Med Res Methodol. 2023 Aug 11;23:182. doi: 10.1186/s12874-023-01997-3 (PMC10422743; doi:10.1186/s12874-023-01997-3)
Supplement: Supplementary file 1 — Additional file 1. [file 12874_2023_1997_MOESM1_ESM.docx]

**Supplementary Appendix**

Table 1

One step prediction differences for a selection of 14 time periods for the overall loss measures: MAPE, MASE (cases) and MAPED and MSED (deaths). The differences are calculated as Base model -Full model.

| Measure | Time point | |  |  |  |  |  |  |  |
| --- | --- | --- | --- | --- | --- | --- | --- | --- | --- |
|  | 20 | 30 | | 50 | 100 | 120 | 145 | 180 | 200 |
| MAPE | -0.029 | -0.051 | | -0.017 | 0.137 | 0.245 | 0.075 | 0.131 | 0.014 |
| MASE | -30.4 | -0.083 | | -0.012 | 0.027 | 0.019 | 0.012 | 0.014 | 0.008 |
|  |  |  | |  |  |  |  |  |  |
| MSED | 2.7E-3 | -0.002 | | 0.001 | -0.003 | 0.001 | -0.075 | -7.7E-3 | -0.003 |
| MAPED | 1.8E-3 | -0.003 | | 9E-3 | -8E-05 | 5.8E-3 | -0.005 | -0.0067 | 3.3E-3 |

| Measure |  |  |  |  |  |  |  |
| --- | --- | --- | --- | --- | --- | --- | --- |
|  | Time point | |  |  |  |  |  |
|  | 250 | 275 | | 300 | 310 | 320 | 350 |
| MAPE | 0.081 | 0.263 | | 0.283 | 0.591 | 0.351 | 0.081 |
| MASE | 0.012 | 0.019 | | 0.019 | 0.041 | 0.021 | 0.003 |
|  |  |  | |  |  |  |  |
| MSED | -0.012 | -0.006 | | 0.131 | 0.014 | -0.003 | 0.041 |
| MAPED | -0.004 | -0.003 | | 0.004 | 0.003 | 7E-3 | 0.008 |
